# Analysis of the complete plastomes of *Bidens pilosa* L. 1753 (Asteraceae, Coreopsideae) from Beijing, China reveals high genetic diversity and possible misidentifications

**DOI:** 10.1080/23802359.2023.2189979

**Published:** 2023-05-31

**Authors:** Xi Wu, Mei Jiang, Michelle Liu, Bin Wang, Qixia Yu, Haimei Chen, Liqiang Wang, Chang Liu

**Affiliations:** a Key Laboratory of Bioactive Substances and Resource Utilization of Chinese Herbal Medicine from the Ministry of Education, Institute of Medicinal Plant Development, Chinese Academy of Medical Sciences, Peking Union Medical College, Beijing, People's Republic of China; b Department of Biomedical Engineering, North Carolina State University, Raleigh, NC, USA; c School of Pharmaceutical Sciences, Xiangnan University, Chenzhou, Hunan Province, People's Republic of China; d College of Pharmacy, Heze University, Heze, Shandong Province, People's Republic of China

**Keywords:** Asteraceae, *Bidens pilosa*, molecular marker, phylogenetic analysis, plastome

## Abstract

*Bidens pilosa* L. 1753 is a perennial herbaceous flowering plant, traditionally used in foods and medicines. In this study, we sequenced, assembled, and characterized the complete plastome of *B. pilosa* from Beijing, China. The plastome (MN385242) is circularized with a conservative quadripartite structure. Its length is 150,524 bp, including a large single-copy region (83,535 bp), a small single-copy region (17,627 bp), and a pair of inverted repeat regions (each 24,681 bp). The plastome consists of 128 genes, including 78 unique protein-coding, 28 unique tRNA, and 4 unique rRNA genes. Phylogenetic analyses showed all five *B. pilosa* plants couldn’t form a monophyletic clade and were separated into three clades. The results of K2P distance and molecular markers were all consistent with those of phylogenetic analysis, revealing high genetic diversity and even possible misidentifications of the *B. pilosa*. Our results highlighted the importance of correct species identification of materials in medicinal products.

## Introduction

*Bidens pilosa* L. 1753 (Bartolome et al. 2013), classified in the Asteraceae, is a perennial and esculent plant. Parts of the plant have been widely used in traditional medicine (Chiang et al. 2003; Andrade-Neto et al. 2004; Sundararajan et al. 2006; Yang et al. 2006). At present, nearly 200 different bioactive compounds have been identified in *B. pilosa*, such as flavonoids, diterpenes, hydrocarbons, and terpenoids (Chang et al. 2004; Wang et al. 2010). These compounds have anti-inflammatory, anti-oxidative, immunomodulatory, and anti-ulcerogenic properties (Bartolome et al. 2013). The pharmacological values of the active ingredients of *B. pilosa* continue to attract research attention.

*Bidens pilosa* is native to America, but it is widely known as an introduced species to other regions of the world. *Bidens pilosa* has six varieties, which are separated into *B. pilosa* var. *pilosa*, var. *minor*, var. *radiata*, var. *bimucronata*, var. *calcicola*, and var. *alausensis* (Sherff 1937) based on their morphological characteristics. In China, *B. pilosa* as a medicinal material has the most commonly used name of ‘Mang-Chang-Cao.’ Two varieties of *B. pilosa* var*. pilosa* and var*. radiata* were described in FRPS (Flora Reipublicae Popularis, http://www.iplant.cn/info/Bidens%20pilosa?t=z). On the medicinal material market, the two varieties are often mixed because of similar morphology. The main difference is that the latter has glossy white flowers, but it is easy to misidentify when it is not flowering or when the glossy flowers abscise.

Alongside morphological traits, the authentication of *B. pilosa* can be aided by chemotaxonomy and molecular characterization (Chien et al. 2009). In a previous study, the DNA barcodes were used to identify three varieties, e.g. *B. pilosa* var. *radiata*, var. *minor,* and var. *pilosa* (Tsai et al. 2008). However, only five loci from the nuclear genome and plastome genome were selected for the variety identification. Complete plastomes with more genetic information are needed for higher species discrimination resolution.

In previous studies, two complete plastomes of *B. pilosa* have been deciphered (Lin et al. 2018; Knope et al. 2020). One of the plastomes was assembled from a specimen from Shaanxi, China (MN729611, labeled as Shaanxi *B. pilosa*), and the other was analyzed from a specimen from Hawaii, USA (MN433104, labeled as Hawaii *B. pilosa*). However, the plastomes from different *B. pilosa* specimens have not been analyzed phylogenetically. In this study, we described a novel phylogenetic relationship for all *Bidens pilosa* specimens and novel molecular markers for sample discrimination.

## Materials

**Figure 1.**
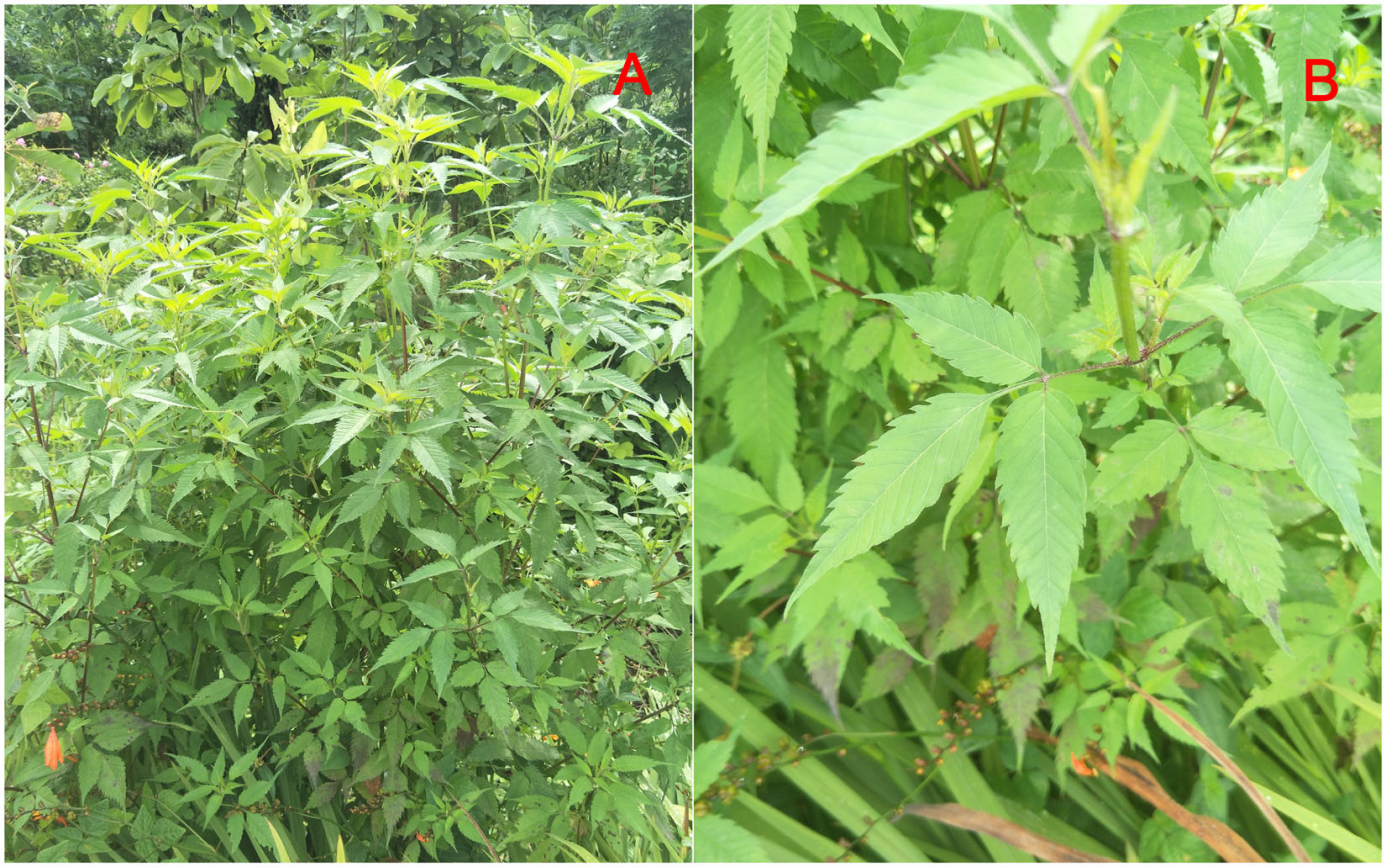
Panorama (A) and detail (B) photos of *Bidens pilosa*. The photos were shot by Liqiang Wang and the coordinates of the plant was E116.278965, N40.041831. Main identifying traits: Capitulum, margin with tongue-like flower 5–7; tongue elliptic obovate, white, 5–8 mm long; apex obtuse or notched.

The *B. pilosa* specimen (Figure 1) were collected from the field of the Institute of Medicinal Plant Development (IMPLAD), Chinese Academy of Medical Sciences, Beijing, China (Geospatial coordinates: E116.278965, N40.041831) and identified by Professor Zhao Zhang of IMPLAD. The voucher samples of the species were deposited in the Herbarium of the institute (voucher number: IMPLADCL150801; contact person: Haimei Chen, hmchen@implad.ac.cn).

## Methods

Total DNA was extracted from fresh leaves using the plant genomic DNA kit (Tiangen, China) and sequenced by the Hiseq 2500 platform (Illumina, Inc., United States). The clean paired-end reads were used to assembly the complete plastome using NOVOPlasty (v. 2.7.2) (Nicolas et al. 2017) with default parameters. The reads were mapped to the assembled genome using Bowtie2 (v.2.0.1) (Langmead et al. 2009) to validate the assembly. The annotation of the plastome was performed using CPGAVAS2 (Shi et al. 2019).

Phylosuite (Zhang et al. 2020) was used to download the GenBank files of the other 45 *Bidens* species plastomes. *Lactuca sativa* and *Taraxacum mongolicum* from Lactuceae were designed as outgroup taxa (Supplementary Table S1). A total of 70 common CDS of the 48 plastome genome were extracted using Phylosuite and aligned using Mafft (Kazutaka and Standley 2013) with default parameters. The aligned CDS sequences were concatenated for reconstructing a Maximum Likelihood phylogenetic tree with the GTR + F + I + G4 model using IQ-tree2 (Quang et al. 2020). The bootstrap support values of the branches were calculated by using UFboot methods with 1000 replicates (Hoang et al. 2018).

## Results

**Figure 2.**
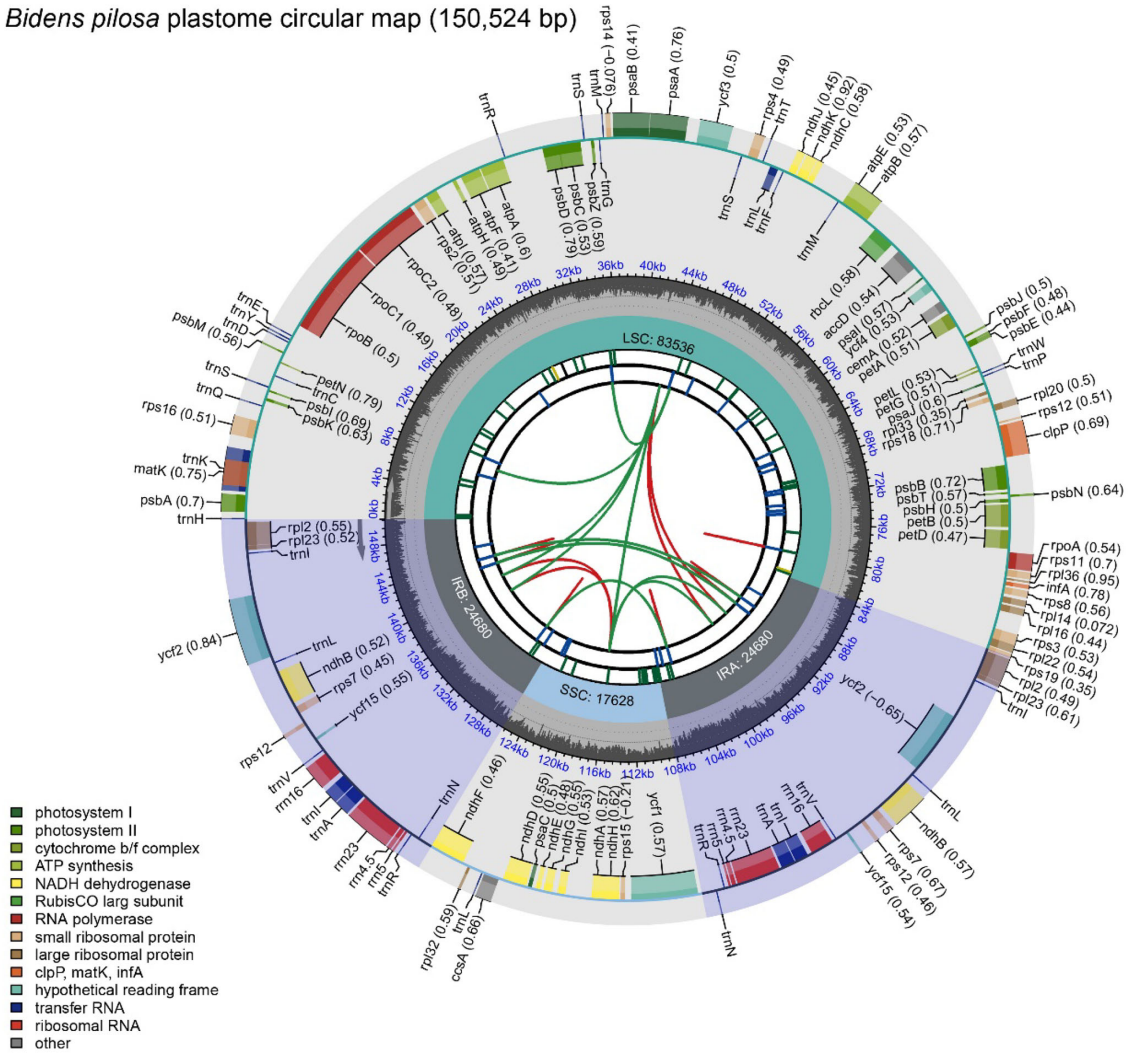
Schematic map of overall features of the *Bidens pilosa* plastome. The map contains six tracks in default. From the center outward, the first track shows the dispersed repeats. The dispersed repeats consist of direct (D) and Palindromic (P) repeats, connected with red and green arcs. The second track shows the long tandem repeats as short blue bars. The third track shows the short tandem repeats or microsatellite sequences as short bars with different colors. The colors, the type of repeat they represent, and the description of the repeat types are as follows. Black: c (complex repeat); Green: p1 (repeat unit size = 1); Yellow: p2 (repeat unit size = 2); Purple: p3 (repeat unit size = 3); Blue: p4 (repeat unit size = 4); Orange: p5 (repeat unit size = 5); Red: p6 (repeat unit size = 6). The small single-copy (SSC), inverted repeat (IRa and IRb), and large single-copy (LSC) regions are shown on the fourth track. The GC content along the genome is plotted on the fifth track. The genes are shown on the sixth track. The optional codon usage bias is displayed in the parenthesis after the gene name. Genes are color-coded by their functional classification. The transcription directions for the inner and outer genes are clockwise and anticlockwise, respectively. The functional classification of the genes is shown in the bottom left corner.

The plastome of the *B. pilosa* deciphered in this study (labeled as Beijing *B. pilosa*) is a circular DNA molecule with a total length of 150,524 bp. The reliability of genome assembly was strongly supported by the results of the mapping experiment (Figure S1). The genome has a conservative quadripartite structure, including a large-single copy (LSC) region, a small-single copy (SSC) region, and a pair of inverted repeats (IR) regions, with a length of 83,535 bp, 17,627 bp, and 24,681 bp, respectively. The GC content is 37.54%, which is lower than that of IR regions (43.06%) and higher than that of the LSC (35.58%) and SSC regions (31.23%). The plastome contains 128 genes, including 78 distinct proteins, 28 distinct tRNAs, and 4 distinct rRNA genes (Figure 2). Seven protein-coding genes (*rps*16, *rpo*C1, *atp*F, *pet*B, *rpl*2, *ycf*2*, ndh*B) contain one intron, and two genes (*ycf3, clp*P) contain two introns. The number of cis-splicing and trans-splicing genes were 11 (*rps*16, *rpo*C1, *atp*F, *ycf*3, *clp*P, *pet*B, *pet*D, *rpl*2, *ndh*B(×2), *rpl*2) and 1 (*rps*12) (Figure S2). Six tRNA genes (*trn*A-UGC, *trn*G-UCC, *trn*I-GAU, *trn*K-UUU, *trn*L-UAA and *trn*C-ACA) contain one intron.

**Figure 3.**
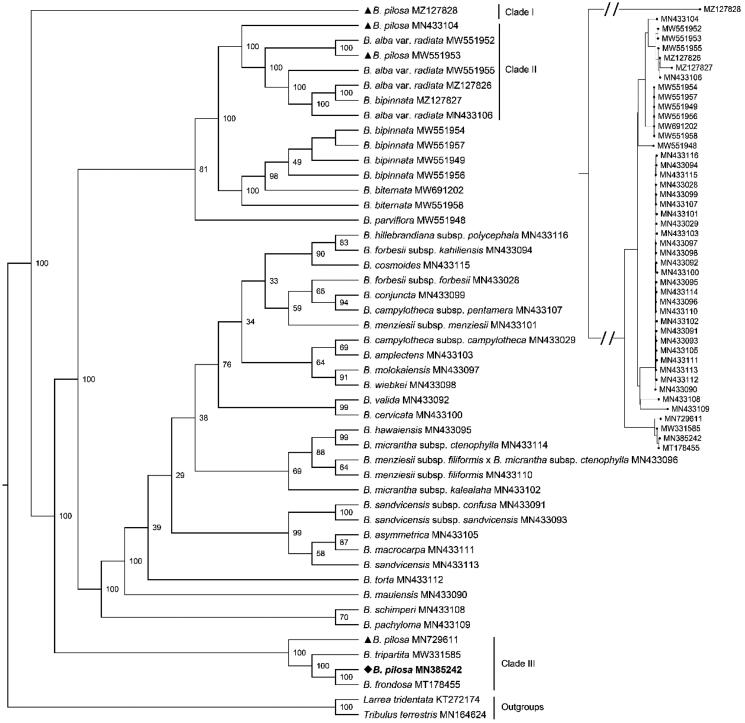
The Maximum-Likelihood phylogeny of *Bidens pilosa* and its close relatives using 70 common CDS sequences. The bootstrap values based on 1000 replicates were shown on each node in the cladogram tree. The corresponding phylogram tree was shown in the upper right corner (Dots represented *Bidens* species). The 45 *Bidens* species Heliantheae alliance were downloaded from GenBank, which were *B. pilosa* (MZ127828), *B. pilosa* (MN433104) (Knope et al. 2020), *B. alba* var. *radiata* (MW551952) (Wu et al. 2022), *B. pilosa* (MW551953) (Wu et al. 2022), *B. alba* var. *radiata* (MW551955) (Wu et al. 2022), *B. alba* var. *radiata* (MZ127826), *B. bipinnata* (MZ127827), *B. alba* var. *radiata* (MN433106), *B. bipinnata* (MW551954), *B. bipinnata* (MW551957), *B. bipinnata* (MW551949), *B. bipinnata* (MW551956), *B. biternata* (MW691202), *B. biternata* (MW551958) (Wu et al. 2022), *B. parviflora* (MW551948) (Wu et al. 2022), *B. hillebrandiana* subsp. *polycephala* (MN433116) (Knope et al. 2020), *B. forbesii* subsp. *kahiliensis* (MN433094) (Knope et al. 2020), *B. cosmoides* (MN433115) (Knope et al. 2020), *B. forbesii* subsp. *forbesii* (MN433028) (Knope et al. 2020), *B. conjuncta* (MN433099) (Knope et al. 2020), *B. campylotheca* subsp. *pentamera* (MN433107) (Knope et al. 2020), *B. menziesii* subsp. *menziesii* (MN433101) (Knope et al. 2020), *B. campylotheca* subsp. *campylotheca* (MN433029) (Knope et al. 2020), *B. amplectens* (MN433103) (Knope et al. 2020), *B. molokaiensis* (MN433097) (Knope et al. 2020), *B. wiebkei* (MN433098) (Knope et al. 2020), *B. valida* (MN433092) (Knope et al. 2020), *B. cervicata* (MN433100) (Knope et al. 2020), *B. hawaiensis* (MN433095) (Knope et al. 2020), *B. micrantha* subsp. *ctenophylla* (MN433114) (Knope et al. 2020), *B. menziesii* subsp. *filiformis* x *B. micrantha* subsp. *ctenophylla* (MN433096) (Knope et al. 2020), *B. menziesii* subsp. *filiformis* (MN433110) (Knope et al. 2020), *B. micrantha* subsp. *kalealaha* (MN433102) (Knope et al. 2020), *B. sandvicensis* subsp. *confusa* (MN433091) (Knope et al. 2020), *B. sandvicensis* subsp. *sandvicensis* (MN433093) (Knope et al. 2020), *B. asymmetrica* (MN433105) (Knope et al. 2020), *B. macrocarpa* (MN433111) (Knope et al. 2020), *B. sandvicensis* (MN433113) (Knope et al. 2020), *B. torta* (MN433112) (Knope et al. 2020), *B. mauiensis* (MN433090) (Knope et al. 2020), *B. schimperi* (MN433108) (Knope et al. 2020), *B. pachyloma* (MN433109) (Knope et al. 2020), *B. pilosa* (MN729611) (Lin et al. 2018), *B. tripartita* (MW331585) (Wu et al. 2022), B. *pilosa* (MN385242, generated in this study, labeled by bold font and a diamond), *B. frondosa* (MT178455) (Feifei Li et al. 2020). Another two species *Lactuca sativa* (KT272174) (Jiang et al. 2021) and *Taraxacum mongolicum* (MN164624) (Kim et al. 2016) from the Lactuceae served as the outgroups. Previous *B. pilosa* plastomes deposited in the GenBank were labeled by triangles. All *B. pilosa* species were located in three clades.

The phylogenetic analysis shown that not all *B. pilosa* plants formed one monophyletic clade (Figure 3). They were separated into three clades. The *B. pilosa* (MZ127828) formed clade I with a bootstrap value of 100, which was the basic branch of the *Bidens* phylogenetic tree. The *B. pilosa* (MW551953), the Hawaii *B. pilosa,* and three *B. alba* var. *radiata* (MW551955, MW551952, and MN433106) formed monophyletic clade II with a bootstrap value of 100. The Beijing *B. pilosa*, the Shaanxi *B. pilosa*, the *B. frondosa* (MT178455), and the *B. tripartita* (MW331585) formed a monophyletic clade III with a bootstrap value of 100. The Beijing *B. pilosa* deciphered in this study was fully resolved on a branch with *B. frondosa* (MW331585).

Subsequently, we calculated the K2P distance between each pair of the five *Biden*s species in clade I, II, and III. We found the K2P distance were from 0.25 to 18.57 (Table S1). The five *B. pilosa* plants was also divided into three clades, which was consistent with that of the phylogenetic analysis. The K2P distance between Beijing *B. pilosa* and *B. frondosa* was 0.02. We further searched the molecular markers between the Beijing *B. pilosa* and the Hawaii plant. Several potential molecular markers can be found between the two plastomes using the ecoPrimer (Riaz et al. 2011) with core parameters of ‘-l 400 -L 500 -e 0 -3 2 -t species -T 1 -U -f -O 25’. Five pairs of conservative sequences were listed in Table 1 with the highest K2P distance which could be used for designing primers for amplifying potential molecular markers.

**Table 1.** Conservative sequences used for designing primers for amplifying potential molecular markers between two *B. pilosa* plastomes.

No.	Genbank accession no.	Forward primer region start-end	Inverse primer region start-end	Sequences for designing forward primers	Sequences for designing inverse primers	Potential molecular markers	K2P distance between two markers
1	MN385242	334–361	408–624	AAACTATGTAAGGCAAATAG TACTAAATAAAAAAAAGGAGCAATA	TATTGCTCCTTTTTTAGTTCAAAAACTCCTA TACAATCAGACCAAAGTCTTATCC ATTTGTAGATGGAGCTTCAATAGCAGCTAAGT CTAGAGGGAAATTATGAGCATTACGTTCATGC ATAACTTCCATACCAAGGTTAGCACGGTTAAT GATATCAGCCCAAGTGTTAATTACACGGCCTT GACTATCAACTACCGATTGGTTGAAATTGAAACC	AAAAAAAAGGAGCAATAGCTTCCCTCT TGATAAAACAAGAGGGCAT	21.02
	MN433104	349–376	423–639			AAAAAAAAGGAGCAATAACG CCCTCTTGTTTTATCAAGAGGGAAGC	
2	MN385242	51,787–51,822	51,892–52,058	CGCTTGATTGTATTTAATCGACAG AATCAAGTCAAA	TTGATAGCCTCTACTCGTGTCCTAGCCCGTCG TAGAGCTAGATTTGCCTCAATTGTTTGTCTCT TTCCTTCAGCTTTTCTCAAAGCATCTTCCGCTA TTTCAAGAGTTTGCTGAGCTTCTTGTGGATCG ATGTCACTACTTTTCTCTGCATCATTTACTAAAACAGT	AAAAACAACCAAACATAGGGT GTATAAATTGAT GATGATTCGGACACACCGACT GGTTATGAAATGGCA	16.54
	MN433104	51,953–51,988	52,071–52,237			CAAAACAACCAAATATAATAATA TAATATAGGGTGTA TAAATTGATGATTATTCGGACAC ACCAACTAGTTATGAAATGGCG	
3	MN385242	64,445–64,495	64,599–64,698	TGATAAGTGAGTTTCTAATAATTCATG AAATGAATTAGAATATTACCGCAA	GAAATTTTCTTCGATAAACGAAAAAGAACAACG AATGAAATAATTGGAATCACTAATGCATGCATT GTTGTATTAGATCGAAATCTGAAATCTGAAGGTT	TTGTAAGTAGATTTGAGATCTATAAATCTTTG TTATTCATAGTCGCTTTTTGTT GGAATCTTTTT TTATCTTTTAAGGAACGTCCAGTA ACAAGGAATAATAG	24.76
	MN433104	64,782–64,832	64,957–65,056			ATTTAAGTAGATTTGAGCTTTGT TATTCATAG TTGTATAAGCGCTTTTTGTTG AAATCTTTTTTT ATCTTTTCTTTTAAGGAATTG GTTTATCTTTTA AGGAATTGGTTAGTCGTCCA GTAACA	
4	MN385242	66,686–66,736	66,779–66,835	AAGGGAGGAAAGAGAAAGAATAAAAGTGCATT CAACAGCTGTGAGACTCGG	AATTTCTATGGAAGTCGAATACAAACAGTGGT TCTAGGGAAAGAGTATTATACAAGA	ATTCAGGTTGGAATTAAATTAAT ATGAAATTTC TATTTATTA	26.35
	MN433104	67,024–67,074	67,119–67,175			GTTCAGTTTGGAATTGAATTAATATT CAATTTCTATGGAAATTC	
5	MN385242	117,415–117,468	117,512–117,589	TATATTCATAATTATATTGATTAGGCGGCGAG AATTGTGTTTTAATTTATATTA	GATGATTTGAAAATATATAGATTCAAATTACTC CTTCGAGAGATTAGGCCAATAACTACATCTAC ATCAATCCATATC	ATATGAAAAATAGATTTCAGTCTATA TTTATTATTTGAAAATT	26.06
	MN433104	117,796–117,855	117,913–117,990			TATGAAAAATTTATTTCTTTTCTATAGT CTATATTGATGATTGATGATTTGAAAATG	

MN385242: Beijing *B. pilosa*. MN433104: Hawaii *B. pilosa*.

## Discussion and conclusion

In this study, we assembled and characterized a complete *B. pilosa* plastome using DNA next-generation data. We performed the phylogenetic analysis, calculated the K2P distance, and searched the molecular markers of different *B. pilosa* plants.

In the phylogenetic analysis, we revealed a large difference among the plastomes of different *B. pilosa* plants. Five *B. pilosa* plants scattered in three separated monophyletic clades, which revealed great variation of different individuals in different habitats or even possible misidentifications of the species. The K2P distance results between each pair of the five *B. pilosa* plants supported the results of the phylogenetic analysis. Furthermore, several potential molecular markers can be searched to distinct the Beijing and the Hawaii *B. pilosa* plant. The above molecular markers indicated the same results that the *B. pilosa* may have large variation among different individuals from different habits or even possible misidentification of the species.

The present results may indicate the *B. pilosa* plants from different habitats have high genetic diversity and even possible misidentifications. In the future, *B. pilosa* samples from various regions should be analyzed, particularly from those materials widely cultivated and used for the manufacturing of medicinal products. Reference markers based on plastomes or nuclear markers of *B. pilosa* will be necessary to exclude misidentifications of this species for the use of the *B. pilosa* materials.

## Supplementary Material

Supplemental Material

Supplemental Material

Supplemental Material

## Data Availability

The plastome sequence has been deposited in GenBank (https://www.ncbi.nlm.nih.gov/genbank/) with the accession number of MN385242 (https://www.ncbi.nlm.nih.gov/nuccore/MN385242). The associated BioProject, Bio-Sample and SRA numbers are PRJNA543381, SAMN16089020 and SRR12620715 (https://www.ncbi.nlm.nih.gov/sra/?term=SRR12620715).
