# Supplementary material for: Analysis of the complete plastomes of Bidens pilosa L. 1753 (Asteraceae, Coreopsideae) from Beijing, China reveals high genetic diversity and possible misidentifications
Source: Mitochondrial DNA B Resour. 2023 May 31;8(5):612–8. doi: 10.1080/23802359.2023.2189979 (PMC10236957; doi:10.1080/23802359.2023.2189979)
Supplement: Supplemental Material [file TMDN_A_2189979_SM6283.docx]

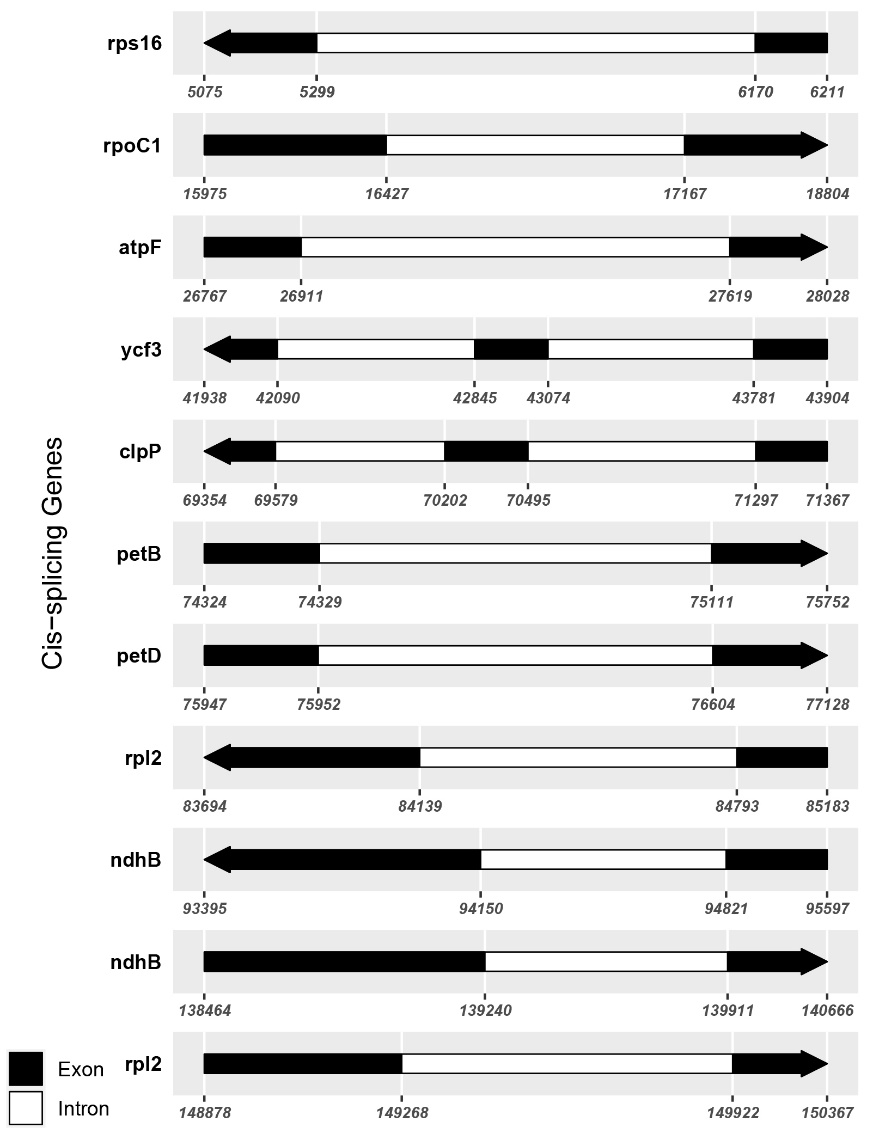


**A**

**B**


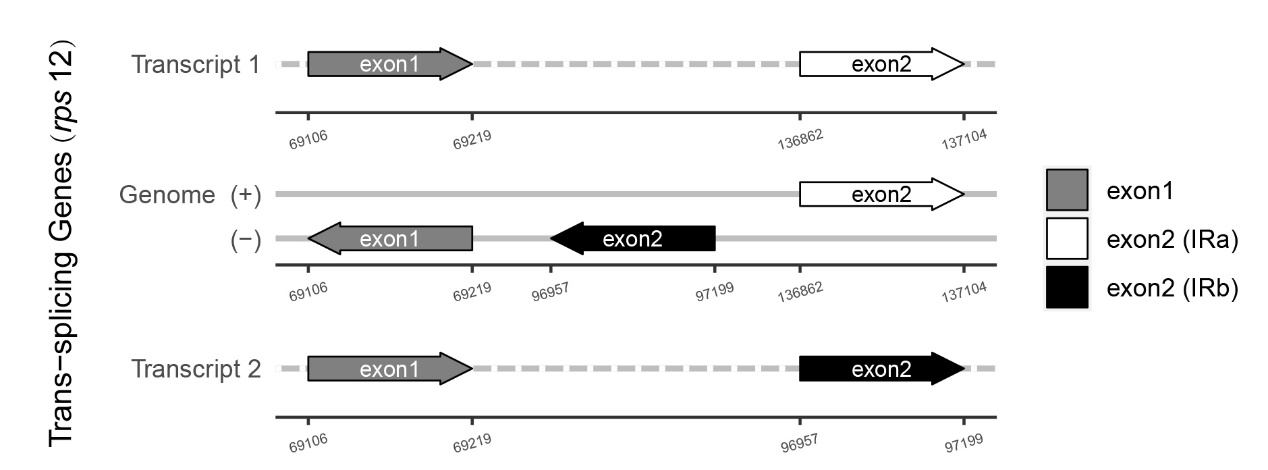


**Figure S2.** The structures of 11 cis-splicing genes (A) and 1 trans-splicing genes (B) of the *Bidens pilosa* plastome.
