# Supplementary material for: Analysis of the complete plastomes of Bidens pilosa L. 1753 (Asteraceae, Coreopsideae) from Beijing, China reveals high genetic diversity and possible misidentifications
Source: Mitochondrial DNA B Resour. 2023 May 31;8(5):612–8. doi: 10.1080/23802359.2023.2189979 (PMC10236957; doi:10.1080/23802359.2023.2189979)
Supplement: Supplemental Material [file TMDN_A_2189979_SM6282.docx]

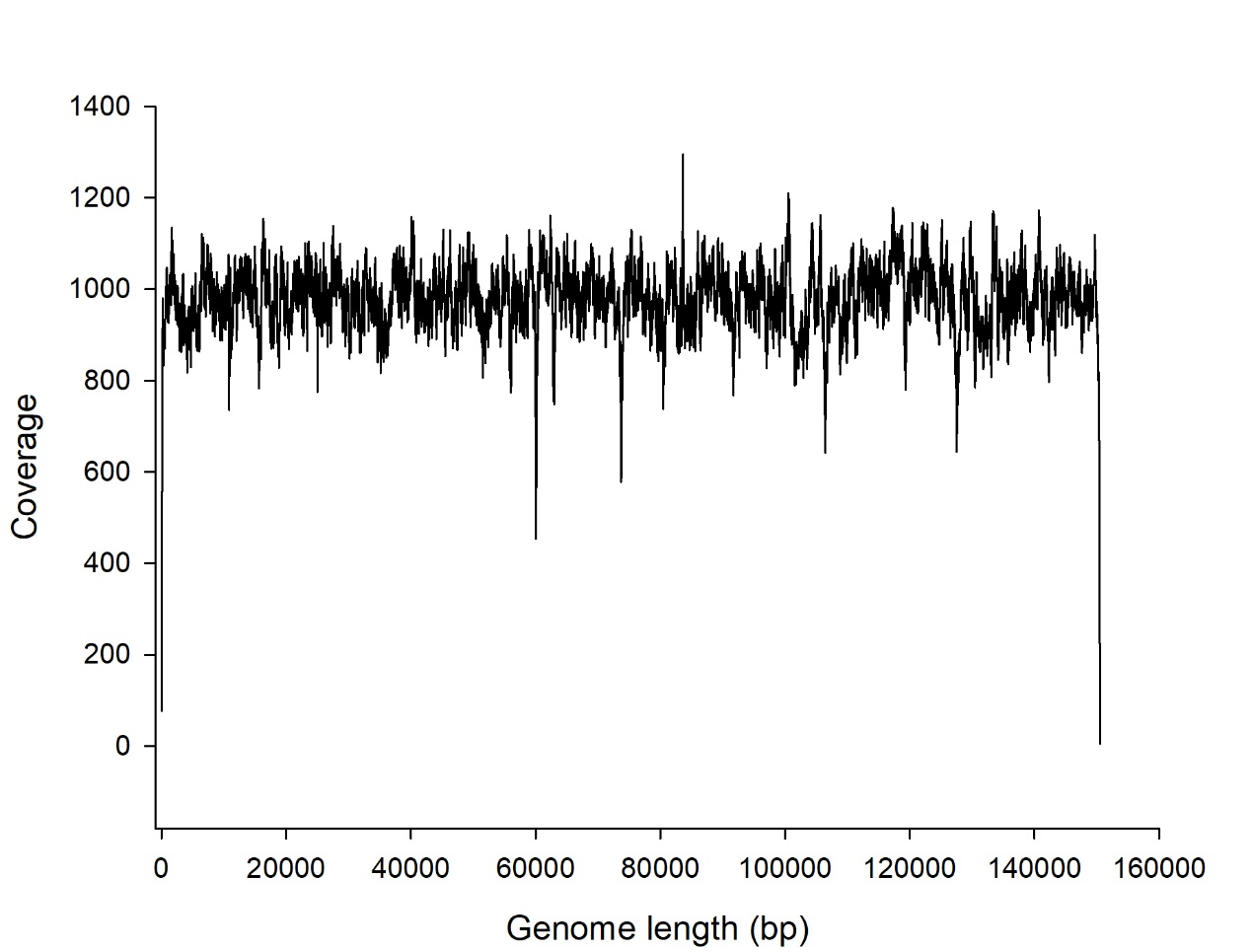


A


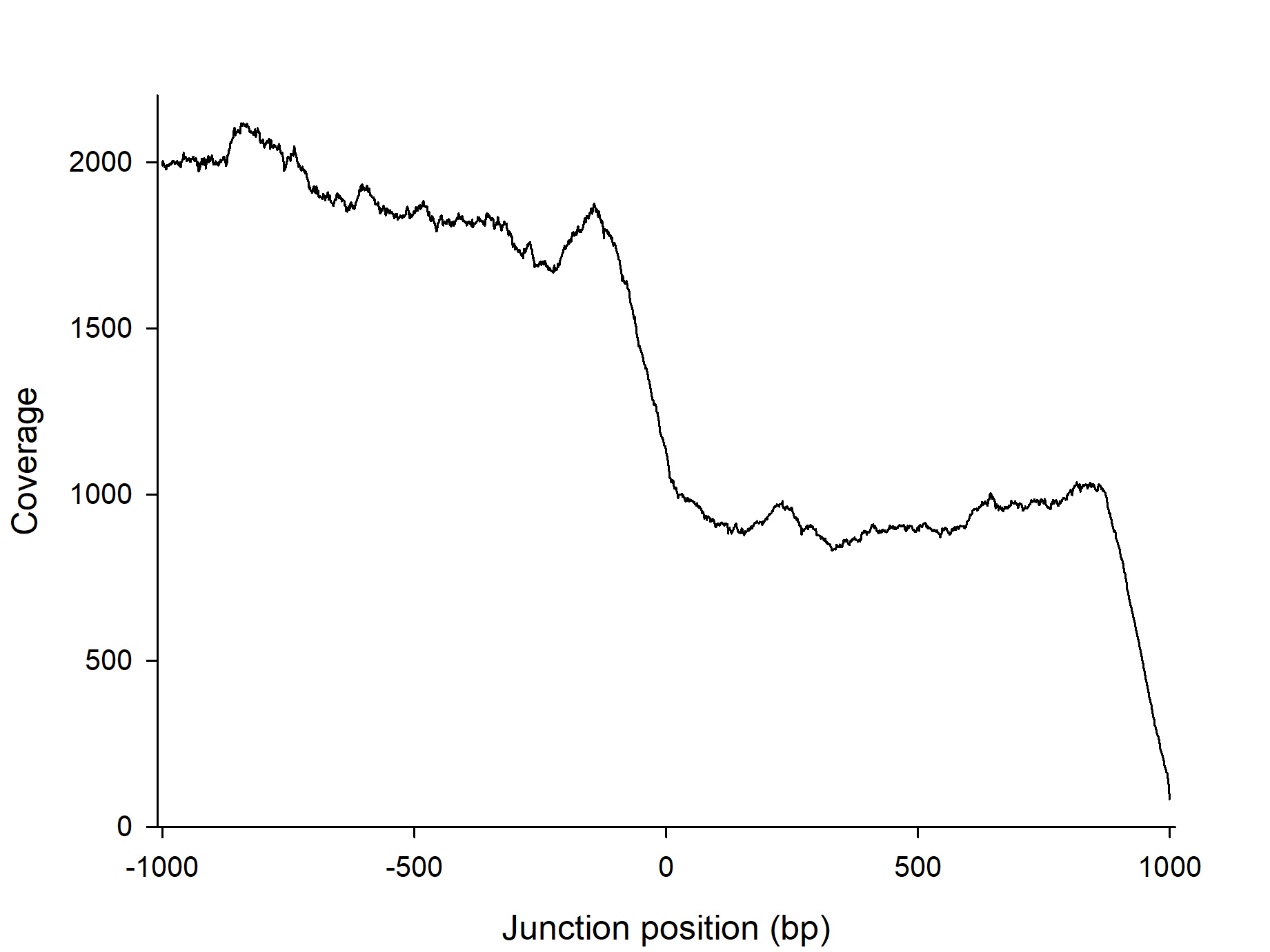


**B**

**Figure S1.** The coverage of the whole assembled *Bidens pilosa* plastome (A) and the junction position of the assembled *Bidens pilosa* plastome (B).
