# Supplementary material for: Analysis of the complete plastomes of Bidens pilosa L. 1753 (Asteraceae, Coreopsideae) from Beijing, China reveals high genetic diversity and possible misidentifications
Source: Mitochondrial DNA B Resour. 2023 May 31;8(5):612–8. doi: 10.1080/23802359.2023.2189979 (PMC10236957; doi:10.1080/23802359.2023.2189979)
Supplement: Supplemental Material [file TMDN_A_2189979_SM6281.docx]

## Table S1. K2P distance between each pair of *Bidens* species in clade I, II and III.

| No. | 1 | 2 | 3 | 4 | 5 | 6 | 7 | 8 | 9 | 10 | 11 | 12 | Accession No. | Latin name |
| --- | --- | --- | --- | --- | --- | --- | --- | --- | --- | --- | --- | --- | --- | --- |
| 1 | 0 | 1.51 | 1.49 | 0.25 | 0.02 | 0.02 | 1.49 | 1.49 | 1.5 | 1.49 | 1.77 | 18.44 | MN385242 | *Bidens pilosa* |
| 2 |  | 0 | 0.41 | 1.59 | 1.52 | 1.52 | 0.41 | 0.41 | 0.42 | 0.41 | 0.75 | 18.55 | MN433104 | *Bidens pilosa* |
| 3 |  |  | 0 | 1.58 | 1.51 | 1.51 | 0.15 | 0.15 | 0 | 0 | 0.34 | 18.52 | MN433106 | *Bidens* *alba* var. *radiata* |
| 4 |  |  |  | 0 | 0.26 | 0.26 | 1.57 | 1.57 | 1.58 | 1.57 | 1.85 | 18.48 | MN729611 | *Bidens pilosa* |
| 5 |  |  |  |  | 0 | 0 | 1.5 | 1.5 | 1.51 | 1.5 | 1.78 | 18.45 | MT178455 | *Bidens frondosa* |
| 6 |  |  |  |  |  | 0 | 1.5 | 1.5 | 1.51 | 1.5 | 1.78 | 18.45 | MW331585 | *Bidens tripartita* |
| 7 |  |  |  |  |  |  | 0 | 0 | 0.14 | 0.15 | 0.49 | 18.5 | MW551952 | *Bidens alba* var. *radiata* |
| 8 |  |  |  |  |  |  |  | 0 | 0.14 | 0.15 | 0.49 | 18.5 | MW551953 | *Bidens pilosa* |
| 9 |  |  |  |  |  |  |  |  | 0 | 0 | 0.35 | 18.52 | MW551955 | *Bidens alba* var*. radiata* |
| 10 |  |  |  |  |  |  |  |  |  | 0 | 0.35 | 18.54 | MZ127826 | *Bidens alba* var. *radiata* |
| 11 |  |  |  |  |  |  |  |  |  |  | 0 | 18.51 | MZ127827 | *Bidens bipinnata* |
| 12 |  |  |  |  |  |  |  |  |  |  |  | 0 | MZ127828 | *Bidens pilosa* |
